# *Salmonella* Subtypes with Increased MICs for Azithromycin in Travelers Returned to the Netherlands

**DOI:** 10.3201/eid2004.131536

**Published:** 2014-04

**Authors:** Robert-Jan Hassing, Wil H.F. Goessens, Wilfrid van Pelt, Dik J. Mevius, Bruno H. Stricker, Nicky Molhoek, Annelies Verbon, Perry J.J. van Genderen

**Affiliations:** Erasmus Medical Centre, Rotterdam, the Netherlands (R.-J. Hassing, W.H.F. Goessens, B.H. Stricker, A. Verbon);; Inspectorate of Health Care, The Hague, the Netherlands (R.-J. Hassing, B.H. Stricker);; National Institute for Public Health and the Environment (RIVM), Bilthoven, the Netherlands (W. van Pelt);; Utrecht University, Utrecht, the Netherlands (D.J. Mevius);; Central Veterinary Institute of Wageningen UR, Lelystad, the Netherlands (D.J. Mevius);; Travel Clinic Havenziekenhuis, Rotterdam (N. Molhoek, P.J.J. van Genderen);; Institute for Tropical Diseases, Havenziekenhuis, Rotterdam (P.J.J. van Genderen)

**Keywords:** Salmonella, typhi, paratyphi, typhoid, bacteria, enteric fever, cephalosporin, fluoroquinolone, azithromycin, drug resistance, travel medicine, the Netherlands

## Abstract

Antimicrobial susceptibility was analyzed for 354 typhoidal *Salmonella* isolates collected during 1999–2012 in the Netherlands. In 16.1% of all isolates and in 23.8% of all isolates that showed increased MICs for ciprofloxacin, the MIC for azithromycin was increased. This resistance may complicate empirical treatment of enteric fever.

Enteric fever caused by *Salmonella enterica* serotypes Typhi and Paratyphi A, B, and C is mainly a disease of the developing world, and it is occasionally diagnosed as an imported disease in countries where the disease is not endemic (*1*). Its empirical treatment has been hampered by resistance to ampicillin, chloramphenicol, and trimethoprim and by decreased ciprofloxacin susceptibility (MIC for ciprofloxacin 0.125–1.0 µg/mL), and ciprofloxacin resistance (MIC for ciprofloxacin >1.0 µg/mL) (*2*,*3*). As a consequence, third-generation cephalosporins are used as first-line drugs for intravenous treatment, and azithromycin is frequently used for empirical treatment of uncomplicated enteric fever.

Although no clinical breakpoints are available to define azithromycin susceptibility or resistance, several clinical studies have demonstrated good efficacy of azithromycin for the treatment of uncomplicated enteric fever in clinical and in vitro studies (*4*–*10*). Regarding MIC breakpoints, the European Committee on Antimicrobial Susceptibility Testing states that isolates with an MIC <16 µg/mL for azithromycin should be considered as wild-type organisms that are responsive to treatment (*3*). The Clinical and Laboratory Standards Institute (www.clsi.org/) does not provide clinical breakpoints for macrolides for the group of *Enterobacteriaceae*. In previous studies of typhoidal *Salmonella* isolates, MICs for azithromycin ranged from 4 µg/mL to 64 µg/mL (*8*–*13*). 

The first clinical case for which treatment of illness caused by typhoidal *Salmonella* spp. with azithromycin (MIC 256 µg/mL) failed was reported during 2010, evidenced by testing a *S. enterica* Paratyphi A isolate from Pakistan (*14*). Further, in a study of isolates from blood samples collected in India during 2005–2008, MICs >16 µg/mL for azithromycin were observed in 34.7% (35/101 isolates) of the typhoidal *Salmonella* isolates; clinical non-response was reported in 19 of 36 patients treated with azithromycin (*13*). Whether this problem of increasing MICs for azithromycin is limited to India or is emerging globally is not clear. The objective of our study was to investigate azithromycin susceptibility and trends in antibacterial drug resistance over time in isolates collected during 1999–2012 in the Netherlands.

## The Study

Enteric fever is a notifiable disease in the Netherlands. During January 1999–December 2012, a total of 354 isolates were submitted by microbiology laboratories to the Salmonella National and Community Reference Laboratory (www.eurlsalmonella.eu/): 177 (50%) *Salmonella* Typhi isolates, 98 (27.7%) *S. enterica* Paratyphi A isolates, 78 (22.0%) *S. enterica* Paratyphi B isolates, and 1 (0.3%) *S. enterica* Paratyphi C isolate. There was no statistically significant difference in sex among patients whose tests showed *S. enterica* Typhi isolates (56.3% male, 43.7% female, p = 0.18) and *S. enterica* Paratyphi isolates (51.6% male, 48.4% female, p = 0.18). Patients ranged in age from 0 to 92 years. The median ages, 28.4 and 29.5 years, respectively (p = 0.60), did not differ between patients in whose samples *S. enterica* Typhi and Paratyphi were isolated. Trends in cumulative 1-year incidence were determined by linear regression analysis; data were weighted on the number of isolates collected each year. The cumulative 1-year incidence of enteric fever was relatively stable during 1999–2012, with an average of 25 isolates (4–39/year) for that period (p = 0.42).

**Figure 1:**
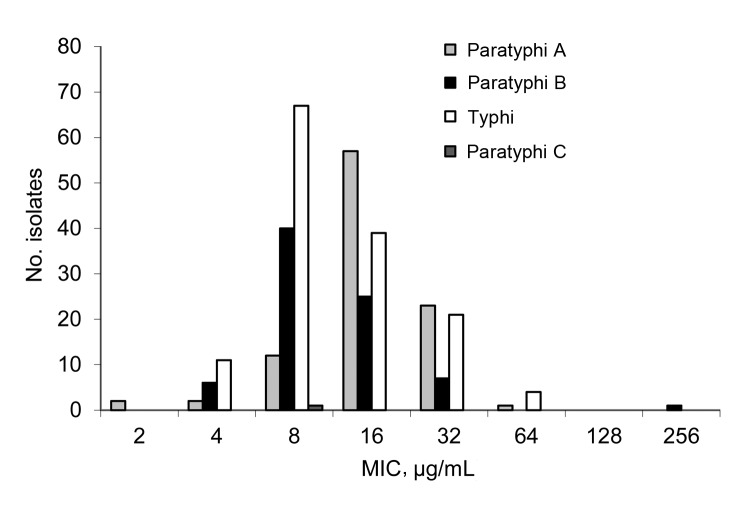
MICs of azithromycin of 354 *Salmonella enterica* serotypes Typhi and Paratyphi A, B, and C isolates from samples collected from ill returned travelers in the Netherlands, 1999–2012. For wild type isolates, MIC<16 μg/mL.

**Figure 2:**
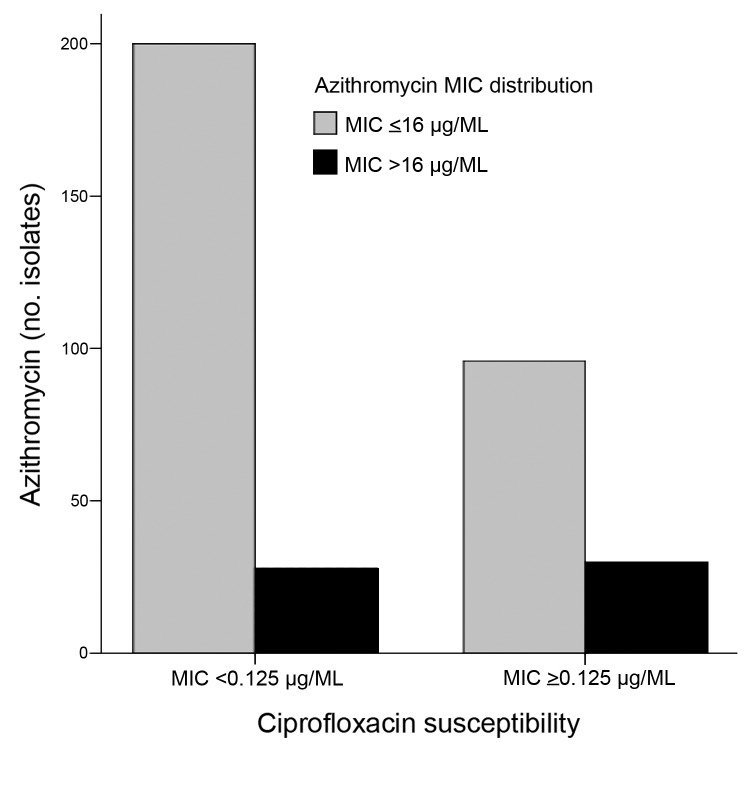
MICs of azithromycin in relation to ciprofloxacin susceptibility of 354 *Salmonella enterica* serotypes Typhi and Paratyphi isolates. Increased MICs for azithromycin (MIC>16 μg/mL) in isolates with decreased ciprofloxacin susceptibility or ciprofloxacin resistance (MIC>0.125 μg/mL) versus ciprofloxacin-susceptible isolates (MIC<0.125 μg/mL) (p = 0.004).

All MICs were determined by using the broth microdilution method with Mueller-Hinton II cation–adjusted broth (Difco, Franklin Lakes, NJ, USA). We applied European Committee for Antimicrobial Susceptibility Testing guidelines for category interpretation for different antibacterial drugs (*3*). Azithromycin MICs were 2–256 µg/mL among the 354 isolates and were increased in 57 (16.1%) of the isolates (Figure 1). The distribution of azithromycin MICs of *S. enterica* Typhi and Paratyphi A and B peaked at 8, 16, and 8 µg/mL, respectively (Figure 1). Trend analysis showed no increased MIC over time for all isolates (p = 0.21) or for *S. enterica* Typhi (p = 0.35) or Paratyphi (p = 0.70). One Paratyphi A isolate, from a sample acquired in Malaysia in 2007, required an MIC of 256 µg/mL. Decreased susceptibility to ciprofloxacin was observed in 116 (32.8%) and ciprofloxacin resistance in 6 (1.7%) of the 354 isolates. Cumulative 1-year incidence of isolates with decreased susceptibility or resistance to ciprofloxacin increased significantly from 0% (0/12 isolates) in 1999 to 64.3% (18/28 isolates) in 2012 (p<0.001) (Technical Appendix Figure). Among isolates with decreased susceptibility or resistance to ciprofloxacin, 23.8% (29/122 isolates) showed an increased MIC for azithromycin; MIC increased for 12.1% (28/232 isolates) of the ciprofloxacin-susceptible isolates (p = 0.004) (Figure 2). No significant increase in amoxicillin, trimethoprim, or chloramphenicol resistance was observed (amoxicillin, p = 0.97; trimethoprim, p = 0.95; and chloramphenicol, p = 0.99) (Technical Appendix Figure). For all isolates, the MICs for erythromycin ranged from 64 to ≥512 µg/mL. Resistance to third-generation cephalosporins was not observed in the isolates.

The origins of isolates for which the country of acquisition was known (205 of 354 strains) were distributed among geographic regions by using the United Nations geoscheme (http://unstats.un.org/unsd/methods/m49/m49regin.htm). Besides imported cases from countries in which enteric fever is highly endemic, such as India, Indonesia, and Pakistan, rates of importation were high for travelers from Turkey and Morocco (Table).

**Table:** Isolates with elevated MICs for ciprofloxacin and isolates with an MIC for azithromycin above that for wild type (>16 mg/L) in *Salmonella enterica* serotype Typhi and Paratyphi isolates collected in travelers returning to the Netherlands, 1999–2012

Region	No. (%) isolates	No. (%) isolates with elevated MICs		
		Azithromycin	Ciprofloxacin	Azithromycin and ciprofloxacin
All	205	36 (17.6)	83 (40.5)	20 (9.8)
Asia	161 (78.5)	31 (19.3)	80 (49.7)	20 (12.4)
Southern Asia*	84 (40.0)	21 (25)	69 (82.1)	18 (21.4)
South-Eastern Asia†	44 (21.5)	5 (12.2)	3 (6.8)	1 (2.3)
Western Asia‡	27 (13.2)	1 (3.7)	6 (22.2)	1 (3.7)
Eastern Asia§	5 (2.4)	1 (20)	2 (40)	0
Unknown	1 (0.5)	0	0	0
Africa¶	35 (17.1)	5 (14.3)	1 (2.9)	0
Europe**	4 (2.0)	0	2 (50)	0
Latin America††	5 (2.4)	0	0	0

*India, Pakistan, Bangladesh, Nepal, Afghanistan, Nepal, Sri Lanka. †Indonesia, Cambodia, Malaysia, Thailand. ‡Turkey, Iraq, Syria. §China. ¶Morocco, Ghana, Nigeria, Senegal, Tunisia, Malawi, Tanzania. **Gibraltar (Great Britain), Greece, Italy, Romania. ††Peru, Mexico, unknown.

Percentages of elevated MICs for azithromycin were highest for isolates acquired in regions that had concurrent high proportions of isolates with decreased susceptibility or resistance to ciprofloxacin (Table). In isolates acquired in countries from Southern Asia, increased MICs for ciprofloxacin and increased MICs for azithromycin were observed in 21.4% (18/84 isolates) of the isolates.

## Conclusions

We found high percentages of elevated azithromycin MICs in typhoidal *Salmonella* isolates collected in the Netherlands during 1999–2012. MICs >16 µg/mL for azithromycin were found in 16.1% of all isolates and in 23.8% of isolates with elevated MICs for ciprofloxacin. This observation may be explained by increased use of azithromycin in countries from which samples yielded high rates of typhoidal *Salmonella* isolates with decreased susceptibility or resistance to ciprofloxacin. Moreover, our findings are aligned with an alarming report from India on increasing MICs for azithromycin (*13*). Our study shows higher MICs than anticipated based on another Western case series (*11*), implying that these potentially resistant strains are not confined to India.

Besides treatment with third-generation cephalosporins, empirical treatment options may be scarce for patients with potential azithromycin-resistant *Salmonella* serotypes. Reuse of antibacterial drugs, such as ampicillin, chloramphenicol, or trimethoprim may be a valuable treatment option upon proven susceptibility, but widespread use of these antibacterial drugs as first-line treatment will likely result in rapid reemergence of multidrug resistance and associated drug-related adverse effects. Further, increasing the dose of ciprofloxacin or using alternative fluoroquinolones has been suggested as an effective treatment in some cases (*15*). This option will not be feasible for empirical treatment because it applies only in a minority of cases and may be associated with drug toxicity. The danger of losing azithromycin to antimicrobial resistance could be detrimental in countries faced with endemic or epidemic enteric fever and complicated by poverty; therefore, azithromycin should be used with care. The results of this study also implicate the importance of developing more effective vaccines as control measures for enteric fever. Future research is needed to evaluate clinically relevant breakpoints of azithromycin by analyzing the treatment outcome of azithromycin in relation to their MICs.

In conclusion, typhoidal *Salmonella* isolates in ill returned travelers from the Netherlands already show a high percentage of increased MICs for azithromycin. Because the highest proportions of increased MICs for azithromycin are found in isolates with increased MICs for ciprofloxacin and in regions where decreased susceptibility or resistance to ciprofloxacin is already widely prevalent among *S. enterica* Typhi and Paratyphi isolates, this resistance may further limit future treatment options for enteric fever.

Technical Appendix Trends in antimicrobial resistance rates of enteric fever isolates of ill travelers returned to the Netherlands, 1999–2012.
