# Supplementary material for: Salmonella Subtypes with Increased MICs for Azithromycin in Travelers Returned to the Netherlands
Source: Emerg Infect Dis. 2014 Apr;20(4):705–8. doi: 10.3201/eid2004.131536 (PMC3966360; doi:10.3201/eid2004.131536)
Supplement: Technical Appendix — Trends in antimicrobial resistance rates of enteric fever isolates of ill travelers returned to the Netherlands, 1999–2012. [file 13-1536-Techapp-s1.pdf]

Article DOI: 10.3201/eid2004.131536

Salmonella Subtypes with Increased MICs for Azithromycin in Travelers Returned to the Netherlands

Technical Appendix

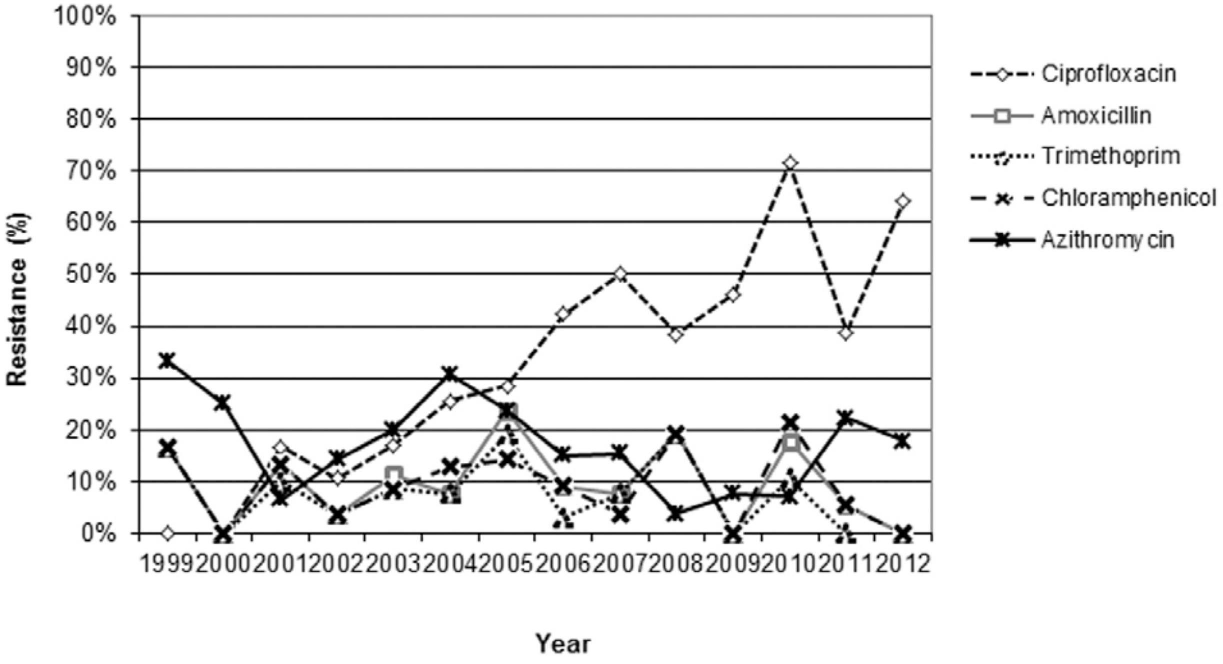

Figure. Trends in antimicrobial resistance rates of enteric fever isolates of ill travelers returned to the Netherlands, 1999–2012. Trend analysis shows significant increase in decreased ciprofloxacin susceptibility or ciprofloxacin resistance (p<0.001).
